# Graves’ Disease and the Heart: Severe Reversible Left Ventricular Systolic Dysfunction With Apical Thrombus in a Young Woman With Relapsed Thyrotoxicosis

**DOI:** 10.7759/cureus.116749

**Published:** 2026-09-22

**Authors:** Jomol Joseph, Chandranath Sarkar, Sai Aditya Boda

**Affiliations:** 1 Internal Medicine, Cardiff and Vale University Health Board, Cardiff, GBR; 2 General (Internal) Medicine, University Hospital of Wales, Cardiff and Vale University Health Board, Cardiff, GBR; 3 Internal Medicine, University Hospital of Wales, Cardiff and Vale University Health Board, Cardiff, GBR

**Keywords:** autoimmune thyroid disorders, graves´disease, heart failure, hyperthyroid cardiomyopathy, hyperthyroidism, left ventricular cardiac thrombus, left ventricular systolic dysfunction, thyrotoxic cardiovascular disease

## Abstract

Graves’ disease is a common cause of autoimmune hyperthyroidism and is frequently associated with cardiovascular manifestations, including sinus tachycardia, atrial fibrillation and high-output heart failure. Severe left ventricular (LV) systolic dysfunction complicated by LV thrombus is an uncommon presentation. Recognition may be challenging when concurrent illness and antithyroid treatment complicate the interpretation of thyroid function tests.

A 27-year-old woman with Graves’ disease diagnosed in 2021 presented in March 2025 with progressive dyspnoea, orthopnoea, profound lethargy, jaundice and generalised oedema. Following relapse in September 2024, carbimazole had been recommenced, but adherence was suboptimal, and heart failure symptoms had developed over several months. Examination revealed sinus tachycardia, diffuse goitre, exophthalmos, hepatomegaly and marked anasarca. Community-acquired pneumonia was also diagnosed. Admission free thyroxine was 8.5 pmol/L (reference range: 8.9-17.3), thyroid-stimulating hormone (TSH) was 0.48 mIU/L (0.30-4.40), and free triiodothyronine was unavailable. This biochemical pattern was interpreted in the clinical context as consistent with non-thyroidal illness.

N-terminal pro-B-type natriuretic peptide (NT-proBNP) was elevated at 9,326 ng/L. Electrocardiography demonstrated sinus tachycardia, and transthoracic echocardiography showed LV dilatation with severe global systolic dysfunction and an estimated LV ejection fraction (LVEF) of approximately 10%. Contrast-enhanced computed tomography demonstrated an LV apical thrombus and hepatic congestion. Graves’ disease-associated myocardial dysfunction was considered an important contributor; however, tachycardia-mediated dysfunction, myocarditis and critical illness-related myocardial dysfunction remained potential contributing causes.

Management included adjustment of carbimazole, diuresis, bisoprolol, valsartan, spironolactone and dapagliflozin, alongside treatment of pneumonia. Therapeutic anticoagulation was initiated with enoxaparin, followed by intravenous unfractionated heparin and bridging to warfarin. Clinical improvement was accompanied by recovery of LVEF to 36% during admission, approximately 40% in June 2025 and 52%-54% on echocardiography in September 2025. Cardiac magnetic resonance imaging during the same time confirmed normal biventricular size and systolic function, with an LVEF of 54% and no residual intracardiac thrombus. A small area of nonspecific non-infarct-pattern fibrosis could represent previous myocarditis. Definitive thyroidectomy was planned.

This case highlights severe but reversible LV systolic dysfunction with apical thrombus in a young woman with relapsed Graves’ disease and suboptimal treatment adherence. Although the clinical course suggests a thyroid-related contribution, the myocardial dysfunction may have been multifactorial. The reduction in thyroid-stimulating immunoglobulins (TSIs) alongside cardiac recovery represents a temporal association and does not establish causation. Careful clinical assessment and cardiac imaging are essential to recognising significant cardiac involvement when thyroid biochemistry is complicated by concurrent illness.

## Introduction

Graves’ disease is a common cause of autoimmune hyperthyroidism and frequently affects young adults. Cardiovascular manifestations range from sinus tachycardia and atrial fibrillation to pulmonary hypertension and heart failure [1,2]. Although thyroid hormone excess typically produces a hyperdynamic circulation, severe left ventricular (LV) systolic dysfunction may occur and can be potentially reversible with appropriate treatment [2,3].

Thyroid hormone excess increases heart rate, myocardial oxygen demand and circulating blood volume while reducing systemic vascular resistance. Prolonged haemodynamic stress and sustained tachycardia may contribute to ventricular remodelling and impaired systolic function [1,3,4]. Understanding the relationship between thyroid disease and cardiac deterioration therefore requires attention to the duration of symptoms, preceding thyroid hormone exposure and treatment adherence, alongside the biochemical findings at presentation.

Hyperthyroidism has also been associated with changes in coagulation and fibrinolysis that favour thrombosis [5,6]. LV thrombus is an unusual feature of Graves’ disease, but severe ventricular systolic dysfunction provides a direct mechanism for its formation through intracavitary blood stasis [7]. This mechanism may operate independently of any additional thyroid-related prothrombotic effect, making the combination of severe LV dysfunction and apical thrombus clinically important.

An autoimmune contribution to myocardial dysfunction in Graves’ disease has been proposed in addition to the haemodynamic effects of thyroid hormone excess. Case-based and myocardial tissue studies have raised the possibility of thyroid-stimulating hormone (TSH) receptor-related autoimmunity, although evidence of myocardial inflammation has been inconsistent [8,9]. The independent contribution of thyroid-stimulating immunoglobulins (TSIs) remains uncertain; their persistence or decline cannot, by itself, establish the mechanism of myocardial injury or recovery.

We report a young woman with relapsed Graves’ disease, suboptimal adherence to carbimazole and gradually progressive heart failure symptoms who presented approximately six months after the documented relapse with severe LV systolic dysfunction and an apical thrombus. Her admission thyroid biochemistry was interpreted as consistent with non-thyroidal illness in the setting of concurrent pneumonia. The subsequent recovery of ventricular function and resolution of the thrombus highlight a potentially reversible presentation and the importance of integrating the longitudinal thyroid history with clinical assessment and cardiac imaging. The case also illustrates the challenges of attributing myocardial dysfunction to thyroid disease when biochemical interpretation is complicated by concurrent illness.

## Case presentation

A 27-year-old woman with Graves’ disease diagnosed in 2021 initially achieved biochemical control with antithyroid therapy. Biochemical relapse was documented in September 2024, and carbimazole 15 mg once daily was prescribed, although subsequent adherence was suboptimal.

Over the months preceding admission in March 2025, she developed progressive dyspnoea, orthopnoea, profound lethargy, generalised oedema, amenorrhoea and jaundice. Examination revealed sinus tachycardia, tachypnoea, bilateral basal crackles, marked exophthalmos, a diffuse goitre, tender hepatomegaly and severe anasarca. Community-acquired pneumonia was diagnosed during the admission.

Thyroid function tests at admission showed a mildly reduced free thyroxine (FT4) with a TSH within reference range; free triiodothyronine (FT3) was unavailable. In the context of severe concurrent illness, these findings were interpreted as consistent with non-thyroidal illness and therefore did not establish euthyroidism. The markedly elevated TSI concentration had been measured approximately six months before admission rather than contemporaneously with the cardiac deterioration.

N-terminal pro-B-type natriuretic peptide (NT-proBNP) was markedly elevated at 9,326 ng/L. Other admission findings included a white blood cell count of 19.1 × 10⁹/L, C-reactive protein of 333 mg/L, bilirubin of 116 µmol/L, and albumin of 26 g/L. Cardiac troponin I was negative. Anticardiolipin and anti-β2-glycoprotein I antibodies were negative, as were liver autoantibodies. Abdominal ultrasonography demonstrated patent portal and hepatic veins without intrahepatic biliary dilatation. Electrocardiography demonstrated sinus tachycardia at approximately 109 beats/minute, without atrial fibrillation on the recorded tracing (Figure 1).

**Figure 1:**
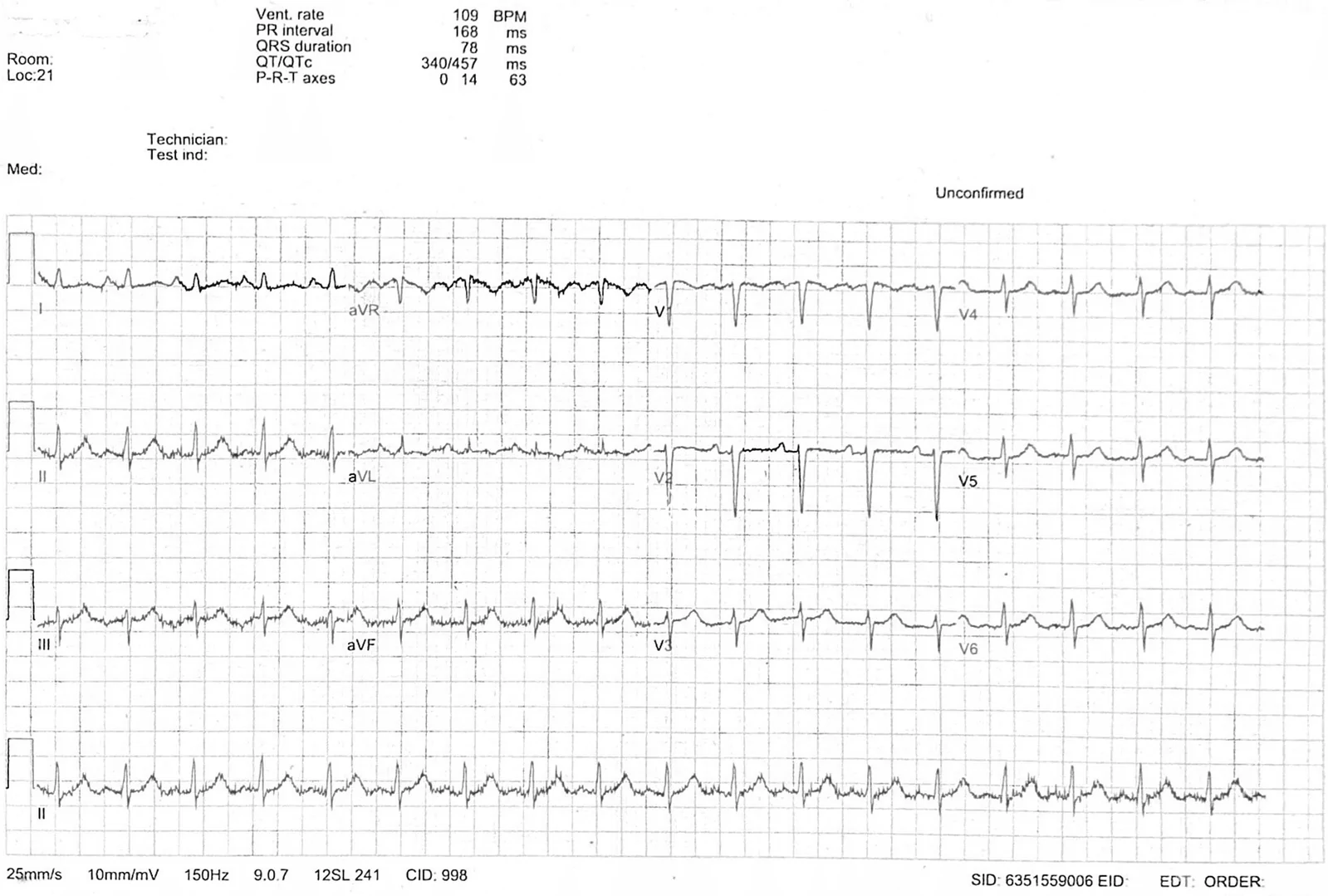
Electrocardiogram at presentation demonstrating sinus tachycardia Twelve-lead electrocardiogram obtained at presentation demonstrating sinus tachycardia at approximately 109 beats/minute. No atrial fibrillation was present on this tracing.

Serial thyroid results and cardiac assessments are summarised in Table 1.

**Table 1:** Thyroid and cardiac biomarkers during disease course TSH: Thyroid-stimulating hormone; TSI: Thyroid-stimulating immunoglobulin; NT-proBNP: N-terminal pro-brain natriuretic peptide; LVEF: Left ventricular ejection fraction.

Investigation (reference range)	September 2024 (Relapse)	March 2025 (Admission)	September 2025 (6 months post-treatment)
Free T3 (2.4–6.0 pmol/L)	10.1	Not available	4.3
Free T4 (8.9–17.3 pmol/L)	7.4	8.5	10.9
TSH (0.30–4.40 mIU/L)	<0.01	0.48	4.13
TSI (<0.55 IU/L)	22.3	Not available	4.85
NT-proBNP (<400 ng/L)	Not available	9,326	Not repeated
LVEF (%)	Not available	10	54

Initial transthoracic echocardiography demonstrated LV dilation and severe global LV systolic dysfunction with an estimated ejection fraction of approximately 10%; an LV mural thrombus was also suspected (Figure 2, Panel A). Contrast-enhanced computed tomography of the thorax, abdomen, and pelvis demonstrated a focal mural low-attenuation filling defect at the LV apex, consistent with LV apical thrombus (Figure 2, Panel B), together with hepatic congestion. Thrombus dimensions and detailed morphological characteristics were not provided in the available reports.

**Figure 2:**
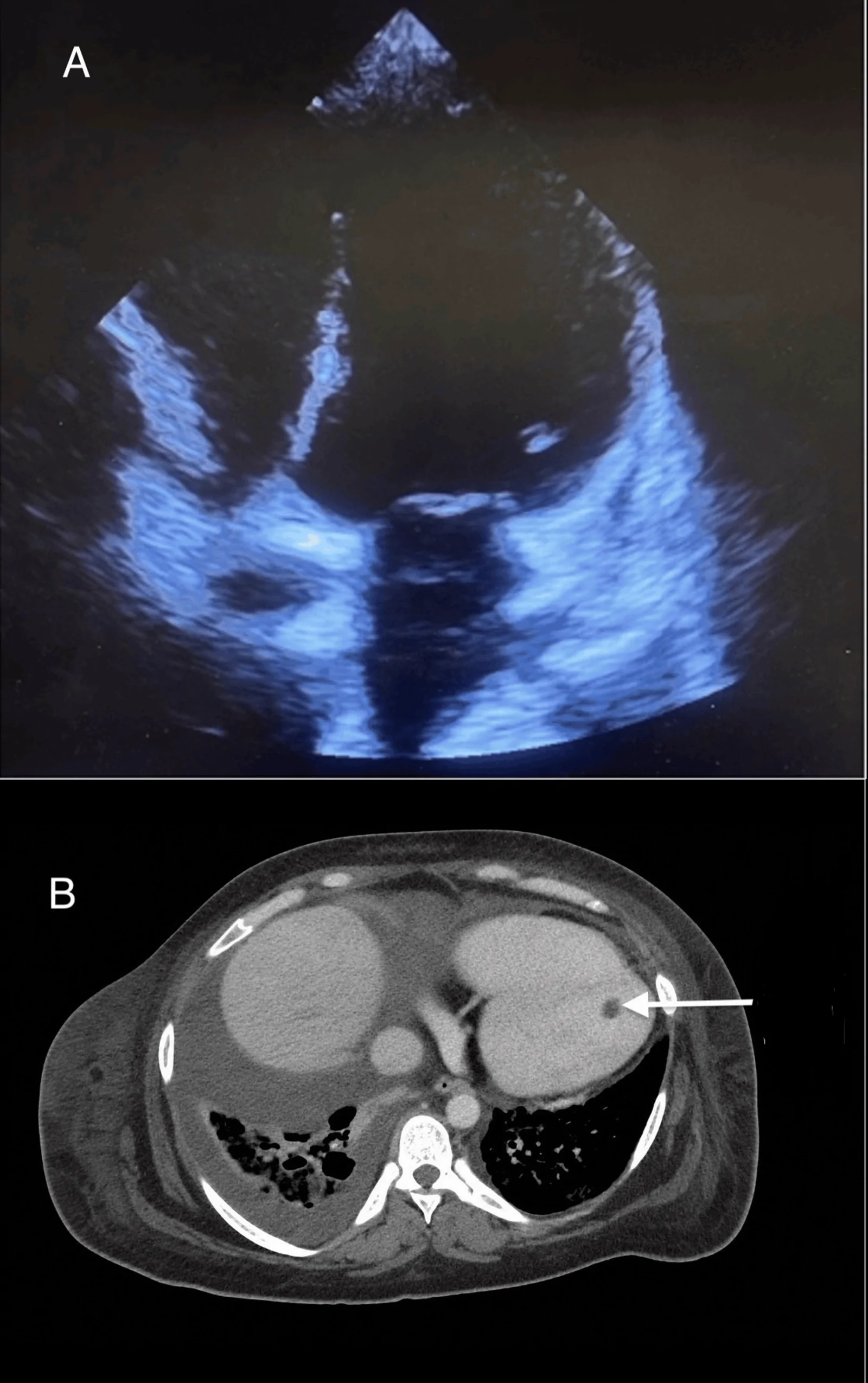
Cardiac imaging at presentation demonstrating severe left ventricular dysfunction and apical thrombus (A) Transthoracic echocardiography at presentation demonstrating left ventricular dilation and severe global left ventricular systolic dysfunction, with an estimated ejection fraction of approximately 10%. (B) Contrast-enhanced computed tomography demonstrating a focal mural low-attenuation filling defect within the left ventricular apex consistent with thrombus; the arrow indicates the left ventricular apical thrombus.

Management

The patient was managed through a multidisciplinary approach involving endocrinology, cardiology, and haematology teams. Community-acquired pneumonia was treated with antibiotics.

Following endocrine assessment, carbimazole was initially withheld in view of the thyroid function tests obtained during the acute illness. It was subsequently restarted at 20 mg once daily and later adjusted according to serial thyroid function tests. Hydrocortisone was administered during the acute illness and subsequently tapered; however, the specific indication was not clearly documented in the available records.

Acute decompensated heart failure with marked fluid overload was initially managed with intravenous furosemide and metolazone, followed by oral diuretic therapy as congestion improved. Guideline-directed medical therapy for heart failure included bisoprolol, valsartan, spironolactone and dapagliflozin, with subsequent adjustment according to clinical status, blood pressure, renal function and tolerability.

Given the confirmed LV apical thrombus in the setting of severe LV systolic dysfunction, therapeutic anticoagulation was initiated to reduce the risk of systemic thromboembolism. Treatment commenced with enoxaparin, followed by intravenous unfractionated heparin and transition to warfarin. Warfarin was continued for approximately six months with INR monitoring. Follow-up echocardiography and cardiac magnetic resonance imaging demonstrated recovery of LV systolic function and no residual intracardiac thrombus, following which anticoagulation was discontinued.

Following clinical stabilisation, ongoing management focused on optimisation of heart failure therapy and control of Graves’ disease. In view of her relapsing Graves’ disease, definitive thyroidectomy was planned.

Outcome and follow-up

The patient demonstrated progressive clinical improvement following treatment, with resolution of orthopnoea and marked improvement in peripheral oedema and other features of congestion. Serial echocardiography in April 2025 demonstrated progressive recovery of LV systolic function, with the LV ejection fraction (LVEF) improving from approximately 10% at presentation to 36% by single plane assessment (Figure 3). The left ventricle remained moderately dilated, and the right ventricle was dilated with severely impaired systolic function. No significant valvular abnormality was reported. Numerical initial ventricular dimensions and pulmonary pressure estimates were unavailable in the retrieved reports. LVEF subsequently improved to approximately 40% in June 2025. Guideline-directed heart failure therapy and warfarin were continued during this period.

**Figure 3:**
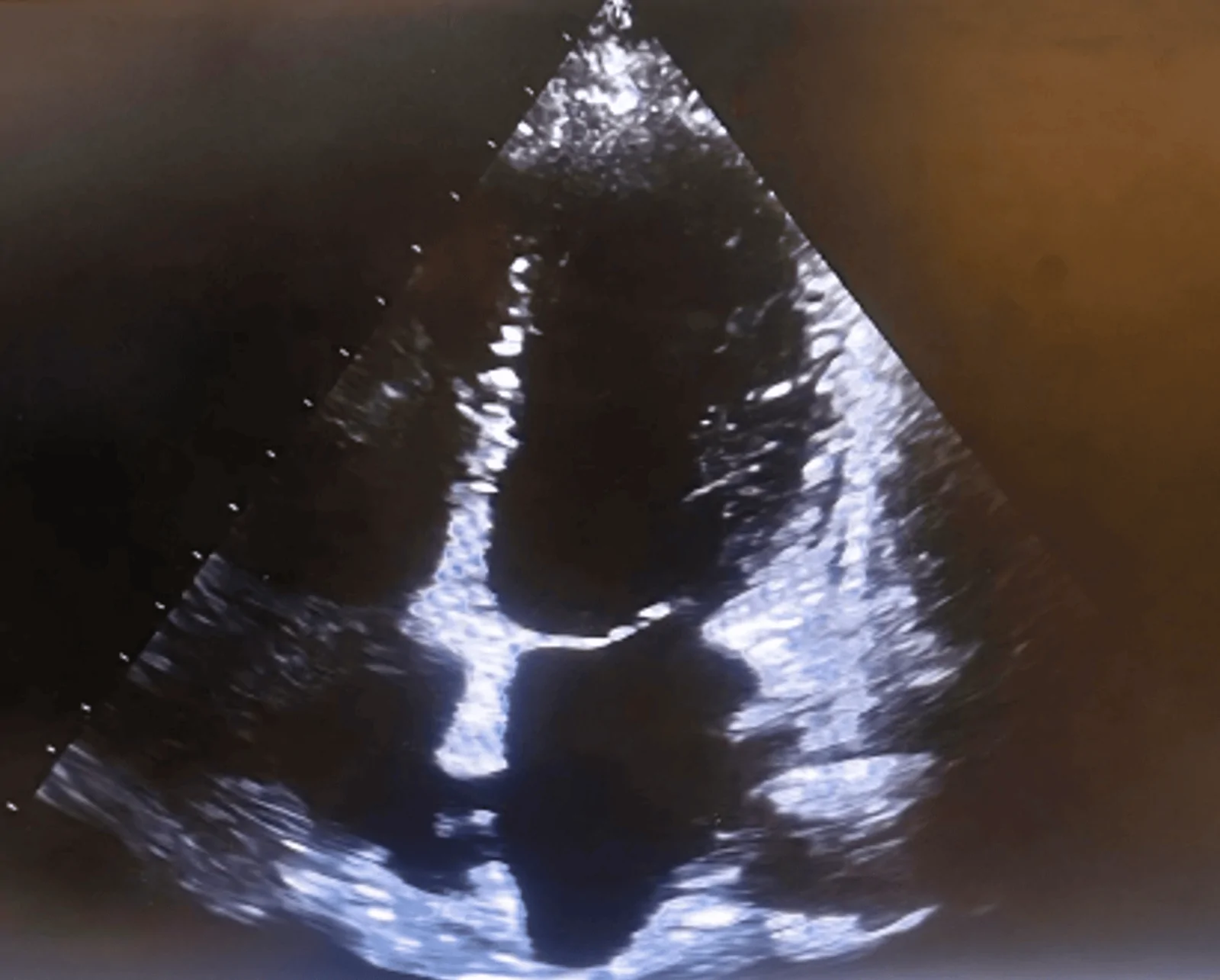
Follow-up echocardiography demonstrating recovery of left ventricular systolic function Transthoracic echocardiography following treatment demonstrating marked improvement in left ventricular systolic function, with the ejection fraction improving from approximately 10% at presentation to approximately 36% following treatment (April 2025).

By approximately six months after treatment, thyroid function had normalised, with FT3 4.3 pmol/L, FT4 10.9 pmol/L, and TSH 4.13 mIU/L. TSI had decreased substantially from 22.3 IU/L at relapse to 4.85 IU/L, although it remained above the reference range. Follow-up echocardiography demonstrated further recovery of LV systolic function, with an LVEF of approximately 52%-54%.

Subsequent cardiac magnetic resonance imaging (CMR) demonstrated normal left and right ventricular size and systolic function, with an LVEF of 54% and right ventricular ejection fraction of 52%. No residual intracardiac thrombus was identified. A small area of focal non-infarct-pattern fibrosis was noted; this was considered nonspecific and could represent previous myocarditis among other aetiologies. Following documented resolution of the LV thrombus, anticoagulation was discontinued.

Ambulatory rhythm monitoring demonstrated sinus rhythm throughout, with a heart rate range of 52-93 beats/minute and no significant arrhythmia. The patient remained under endocrinology and cardiology follow-up, with definitive thyroidectomy planned.

Overall, the clinical course demonstrated substantial recovery of LV systolic function alongside biochemical control of Graves’ disease and resolution of the LV thrombus. The preceding biochemical relapse of Graves’ disease, suboptimal adherence to antithyroid therapy, persistent autoimmune activity reflected by the markedly elevated TSI and subsequent improvement in both thyroid parameters and cardiac function following treatment support an important thyroid-related contribution to the severe LV systolic dysfunction. However, a direct causal relationship cannot be established, and the LV dysfunction was likely multifactorial. Concurrent severe systemic illness and the possibility of previous myocarditis, suggested by the nonspecific CMR finding, may also have contributed to the initial cardiac dysfunction.

The overall clinical course, including the initial diagnosis of Graves’ disease, subsequent relapse, development of severe LV systolic dysfunction and LV thrombus, recovery of ventricular function, thrombus resolution and planned definitive thyroid treatment, is summarised in Figure 4.

**Figure 4:**
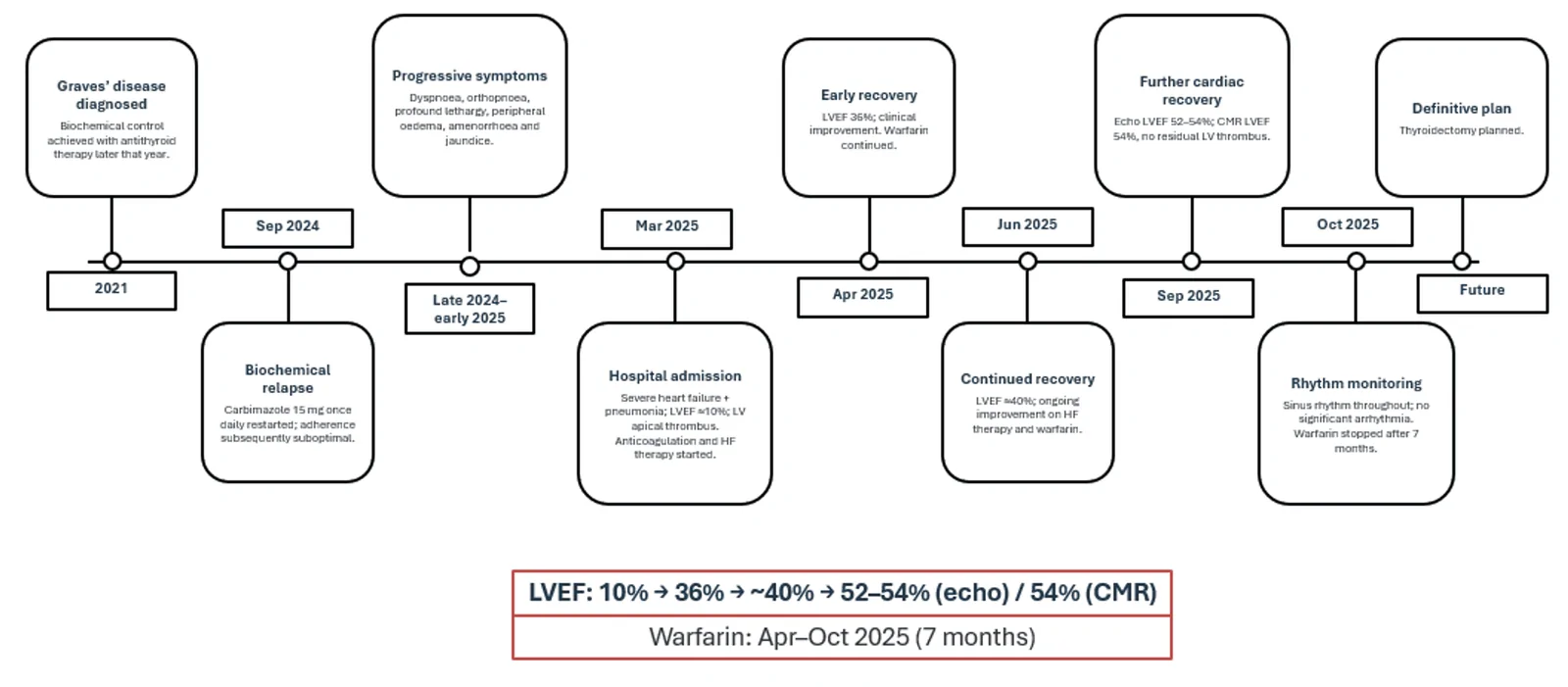
Timeline of clinical course and cardiac recovery Timeline illustrating the clinical course from the initial diagnosis of Graves’ disease through relapse, development of severe left ventricular systolic dysfunction and left ventricular thrombus, subsequent recovery of left ventricular function, thrombus resolution and planned definitive thyroidectomy. Credits: This image was created by the authors using Microsoft PowerPoint (Microsoft Corp., Redmond, WA). LVEF: Left ventricular ejection fraction; LV: Left ventricle; CMR: Cardiac magnetic resonance; INR: International normalised ratio.

## Discussion

This case demonstrates severe, subsequently reversible LV systolic dysfunction complicated by an apical thrombus in a young woman with relapsed Graves’ disease. The recovery of ventricular function and resolution of the thrombus are the principal clinical observations. Although the thyroid history suggests an important contribution from Graves’ disease, the available findings support a potentially multifactorial interpretation rather than a definitive attribution to thyrotoxicosis alone.

The documented relapse, suboptimal adherence to carbimazole and progressive heart failure symptoms over several months provide a plausible clinical basis for thyroid-related myocardial dysfunction. Prolonged thyroid hormone excess can increase myocardial workload through tachycardia, increased circulating blood volume and altered systemic vascular resistance, potentially leading to ventricular remodelling and systolic impairment [1-4]. However, intermittent biochemical measurements and uncertainty regarding medication adherence limit assessment of the duration and severity of thyroid hormone exposure in this patient.

The admission thyroid results require particular caution. FT4 was below the laboratory reference range, TSH was within range and FT3 was unavailable. These findings were interpreted clinically as consistent with non-thyroidal illness in the setting of concurrent pneumonia, but they did not establish euthyroidism or reliably characterise the preceding thyroid state. Furthermore, FT3 had been elevated during the September 2024 relapse despite a low FT4 concentration, making comparison of FT4 alone an inadequate measure of biochemical improvement. 

Underlying autoimmunity provides a further hypothesis, although the supporting evidence is limited. TSI was markedly elevated at the documented relapse and remained above the reference range on subsequent testing. Experimental observations and case-based evidence have suggested a possible role for TSH receptor-related autoimmunity in myocardial involvement, while endomyocardial biopsy findings have not consistently demonstrated an inflammatory process [8,9]. In this patient, TSI was not measured at cardiac presentation, so the earlier elevation cannot establish contemporaneous autoimmune myocardial injury. Its subsequent decline alongside ventricular recovery represents a temporal association. Because antithyroid treatment, heart failure therapy and treatment of the concurrent illness were administered together, the independent contribution of an autoimmune mechanism cannot be determined.

The concurrent pneumonia and marked inflammatory response also complicate diagnostic attribution. Severe systemic illness may have contributed to cardiac decompensation or aggravated pre-existing myocardial dysfunction. The gradual development of symptoms before admission suggests that the acute infection may not fully explain the clinical course, but an infection-related contribution cannot be excluded.

Myocarditis remained another possible contributor. Follow-up cardiac magnetic resonance imaging demonstrated a small area of nonspecific subepicardial, non-infarct-pattern fibrosis that could represent previous myocarditis. This finding did not establish the cause or timing of myocardial injury, but it prevented confident exclusion of a previous inflammatory process. A negative admission troponin I result does not independently exclude myocarditis [10]. Moreover, the MRI was obtained after ventricular recovery and could not fully characterise the acute myocardial process. Dedicated coronary imaging was not documented; consequently, an ischaemic aetiology was not definitively excluded.

A tachycardia-mediated component also could not be established or excluded. The admission electrocardiogram showed sinus tachycardia at approximately 109 beats/minute without atrial fibrillation. This single recording did not quantify the preceding duration or burden of tachycardia. Although later Holter monitoring demonstrated sinus rhythm without significant arrhythmia, the absence of prolonged rhythm assessment during the acute presentation limits retrospective assessment of this mechanism.

The LV apical thrombus is an important feature of the case. Severe global systolic dysfunction provides a strong mechanism for thrombus formation through intracavitary blood stasis [7]. Hyperthyroidism-associated changes in coagulation and fibrinolysis may have provided an additional contribution, although their role in this patient cannot be demonstrated [5,6]. Negative anticardiolipin and anti-β2-glycoprotein I antibodies provided no support for these antiphospholipid antibodies as an explanation but did not establish exclusion of every thrombophilic disorder. Thrombus resolution was subsequently documented by cardiac MRI following anticoagulant treatment and improvement in ventricular function.

Overall, the clinical course supports potentially reversible myocardial dysfunction in the setting of relapsed Graves’ disease, with possible contributions from preceding thyroid hormone exposure, tachycardia, systemic illness and myocardial inflammation. Recovery during combined treatment cannot distinguish the relative contribution of each mechanism. The case emphasises the value of a longitudinal thyroid history, assessment of treatment adherence and serial cardiac imaging, while illustrating the need to distinguish observed clinical recovery from an unproven causal explanation.

## Conclusions

This case demonstrates substantial recovery of severe LV systolic dysfunction and resolution of an apical thrombus in a young woman with relapsed Graves’ disease receiving combined thyroid, heart failure and anticoagulant treatment. The preceding relapse, suboptimal medication adherence and gradual symptom progression suggest a thyroid-related contribution. However, concurrent infection, possible previous myocarditis and an incompletely characterised tachycardia burden support a potentially multifactorial explanation. Admission thyroid biochemistry, interpreted as consistent with non-thyroidal illness, did not establish euthyroidism. Similarly, the decline in TSIs alongside cardiac recovery does not establish an independent autoimmune mechanism.

The case highlights the importance of integrating the longitudinal thyroid history and treatment adherence with clinical assessment and serial cardiac imaging. Coordinated multidisciplinary care was accompanied by marked improvement, while continued endocrine and cardiac follow-up remained important for assessing recovery and planning definitive management of relapsed Graves’ disease.
